# Construction of immune cell infiltration protein network based on clinical low grade glioma cases

**DOI:** 10.3389/fonc.2022.956348

**Published:** 2022-09-20

**Authors:** Wei Jiang, Zijian He, Weizhong Jiang, Jiarui Du, Lutao Yuan, Cong Luo, Xiang Li, Fulin Xu

**Affiliations:** ^1^ Department of Neurosurgery, Minhang Hospital, Fudan University, Shanghai, China; ^2^ Department of Critical Care Medicine, Minhang Hospital, Fudan University, Shanghai, China

**Keywords:** glioma, germinal signal analysis, protein network, immune infiltration, PIN (protein interaction network)

## Abstract

Many researchers have studied low-grade glioma and the immune microenvironment have been studied by many researchers. Recent studies suggest that macrophages and dendritic cells trigger part of the local immune dysregulation in the tumor microenvironment, and they have been polarized into a mixed pro-inflammatory and immunosuppressive phenotype. It is suggested that the degree of immune infiltration is related to the survival, therapeutic effect, and prognosis of patients. This opens up new avenues for cancer treatment. On the basis of immune infiltration degree, a protein interaction network (PIN) and a prognosis model were established, and we chose the top 20 pathways from enrichment analysis to provide potential targets for glioma clinical treatment.

## 1 Introduction

According to the World Health Organization (WHO) classification of tumors of the central nervous system, gliomas can be classified into four grades as the most common major brain tumors worldwide (1–5). Twenty percent of gliomas are low-grade gliomas (LGG), with various clinical manifestations (6, 7), the prognosis can be relatively good (8–10). Current treatment for LGGs tends to favor maximum excision at an early stage, with consideration of combined radio-chemotherapy for “high-risk” patients (11). However, after conventional treatment, the 10-year survival rate of LGG patients remains unchanged at less than 50% (12, 13).

Recent studies indicate that macrophages and dendritic cells are motivated by immune dysregulation in the tumor microenvironment, and they differentiate into a mixed proinflammatory/immunosuppressive phenotype (14, 15). These results suggest that there may be a relationship between tumor invasion and survival, treatment effect, and prognosis, and this relationship may provide a new method for tumor treatment.

In this study, we obtained 529 glioma cases from public data TCGA and investigated the prognosis, clinical traits, and immunotherapy. We calculated the degree of immune cell infiltration and divided them into two groups using The Cancer Genome Atlas Low Grade Glioma (TCGA-LGG) as the standard to determine the different genes. Then, we performed a functional enrichment analysis of pathogenic proteins associated with LGG, constructed a protein interaction network, identified densely connected network components, and found the top 20 pathways. The study aims to find the classical protein interaction pathway through analysis and present novel perspectives and aims for future LGG clinical treatment.

## 2 Methods and results

### 2.1 Experimental methods: Raw data acquisition and analysis

Data of 529 tumor cases were obtained from the TCGA, and the prognosis, clinical traits, as well as immunotherapy were investigated, and the degree of immune cell infiltration of 22 immune cells of 529 tumor samples of the TCGA-LGG [(fpkm) PKM] was calculated by CIBERSORT, TCGA-LGG was divided into two groups by the degree of immune cell infiltration using the “consensusclusterplus” R package to investigate their correlation with prognosis, clinical traits, as well as immunotherapy. The TCGA-LGG (count) was used with edger to determine the differential genes of these two groups (*p <*0.01, logfc >1.5). Enrichment analysis was performed using Metascape, and the PPI protein interaction network was constructed to find the densely connected network components, and the top 20 pathways according to p-value sorting for subsequent study. The differential expression and existence of specific genes in LGG were confirmed by Western blot.

### 2.2 Analysis of outcomes

#### 2.2.1 Analysis of different immune cell species

**Figure 1:**
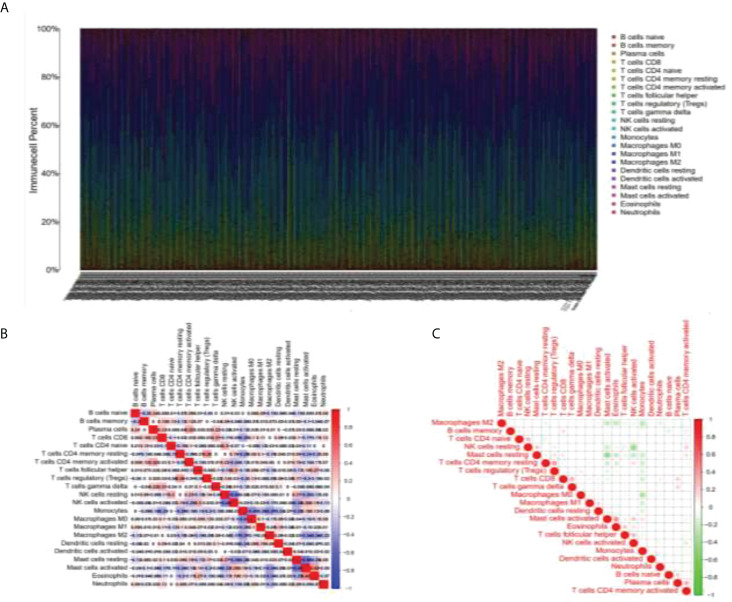
Immunocyte analysis in LGG samples. **(A)** Figure line of immune cell content in different LGG samples. **(B, C)** The selected immune cells were subjected to Spearman analysis and a correlation analysis of immune factors was carried out.

As shown in **Figure 1A**, the abscissa shows different LGG samples, the ordinate is the percentage of immune cell content, and different colors indicate different immune cell species. A total of 22 immune cells were studied, and the 22 immune cells screened were subjected to Spearman analysis and classified into two groups according to different microenvironmental infiltrates. In **Figures 1B, C**, the analysis indicates that many immune cells such as Tregs, CD8+ T cells, plasma cells, and B cells are positively related to LGG, NK, and mast cells are negatively related.

#### 2.2.2 A total of 529 LGG patients were grouped

**Figure 2:**
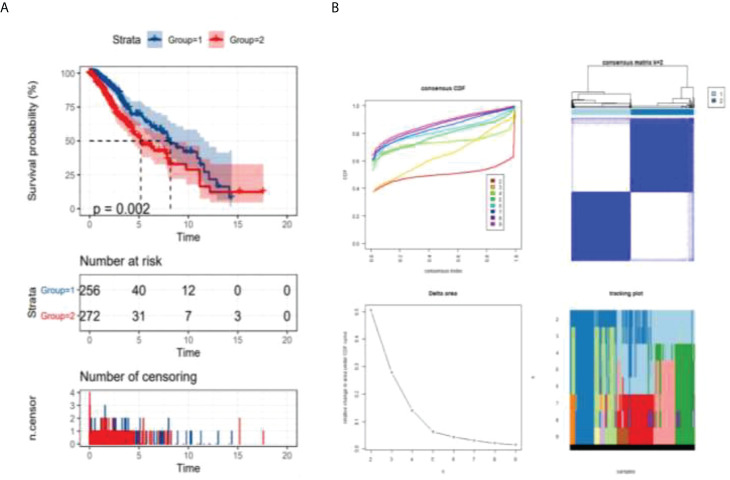
Analysis of survival probability of LGG patients. *p* = 0.002 < 0.05 (logrank test) **(A)** survival analysis of different groups, **(B)** consensus analysis defined two possible groups.

This study separated all patients with LGG into two groups by consensusclusterplus based on the immune cell microenvironment infiltrated differently and performed survival probability analysis. As shown in **Figure 2A**, the red curve represents the cluster 2 group survival curve, and the blue curve represents the cluster 1 group survival curve. P-value = 0.002 after the logrank test, indicating that we were unable to use sampling error to explain the difference in survival status between the two groups and that grouping factors were responsible for the difference in survival between the two curves. This example graphically shows that overall survival was better in the cluster 1 group than in the cluster 2 group. As shown in consensus analysis defifined two possible groups (**Figure 2B**).

#### 2.2.3 Immune cell analysis by cluster 1 and cluster 2 distinct

**Figure 3:**
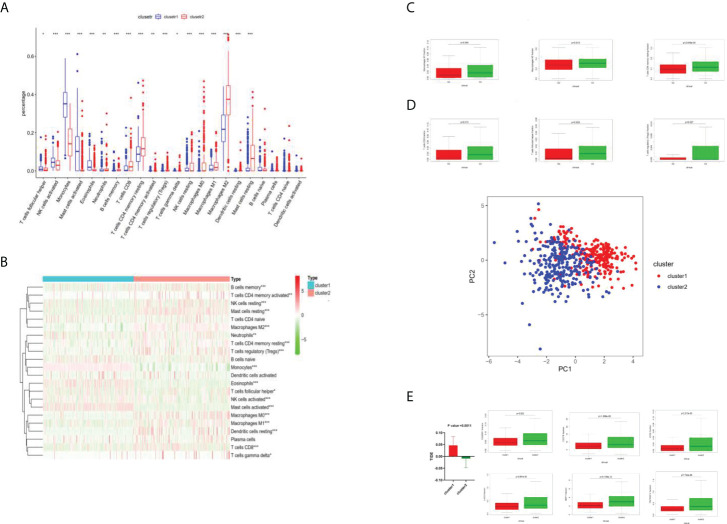
Immune cell analysis by cluster 1 and cluster 2. **(A)** Boxplots of immune cell difference analysis. *P <0.05; **P <0.01; ***P <0.001. **(B)** Heat diagram of different degrees of immune cell infiltration between cluster 1 and cluster 2. **(C)** Different levels of immune cell infiltration were different in the G2 and G3 phases. **(D)** The degrees of immune cell infiltration of all samples were analyzed by principal component analysis, and the above clustering can be well divided into two categories. **(E)** TIDE value analysis.

As shown in **Figure 3A**, different immune cell infiltration degrees are associated with differences in G2 and G3 stages. The Wilcox test received a p-value. All P-values were less than 0.05, which indicated that the difference in the G2–G3 phase was statistically significant. As shown in **Figure 3**, differential analysis of immune cells between cluster 1 and cluster 2 was performed according to the boxplots (*p <0.05; **p <0.01; ***p <0.001.): both clusters 1 and 2 showed differential immune cell infiltration in the illustrated species, with the most significant differences being for macrophage M2, monocytes, and resting memory CD4 T cells (**Figure 3B**). Where differences in immune cell infiltration were known, immune check site genes were analyzed for expression in cluster 1 and cluster 2, and the Wilcox test yielded a p-value (**Figure 3C**).The degree of immune cell infiltration of all samples was analyzed by principal component analysis, and the above clustering can be well divided into two categories (**Figure 3D**). In **Figure 3E**, TIDE was used to predict the efficacy of immunotherapy for all samples, and the TIDE value of each sample was obtained. Red indicates the mean ±95% CI of cluster 1, green indicates the mean ±95% CI of cluster2. The T-test showed that P =0.0011. The higher the TIDE value is, the less sensitive it is to immunotherapy such as PD1; the lower the TIDE value is, the more sensitive it is.

#### 2.2.4 GO and KEGG analysis of related gene pathways

**Figure 4:**
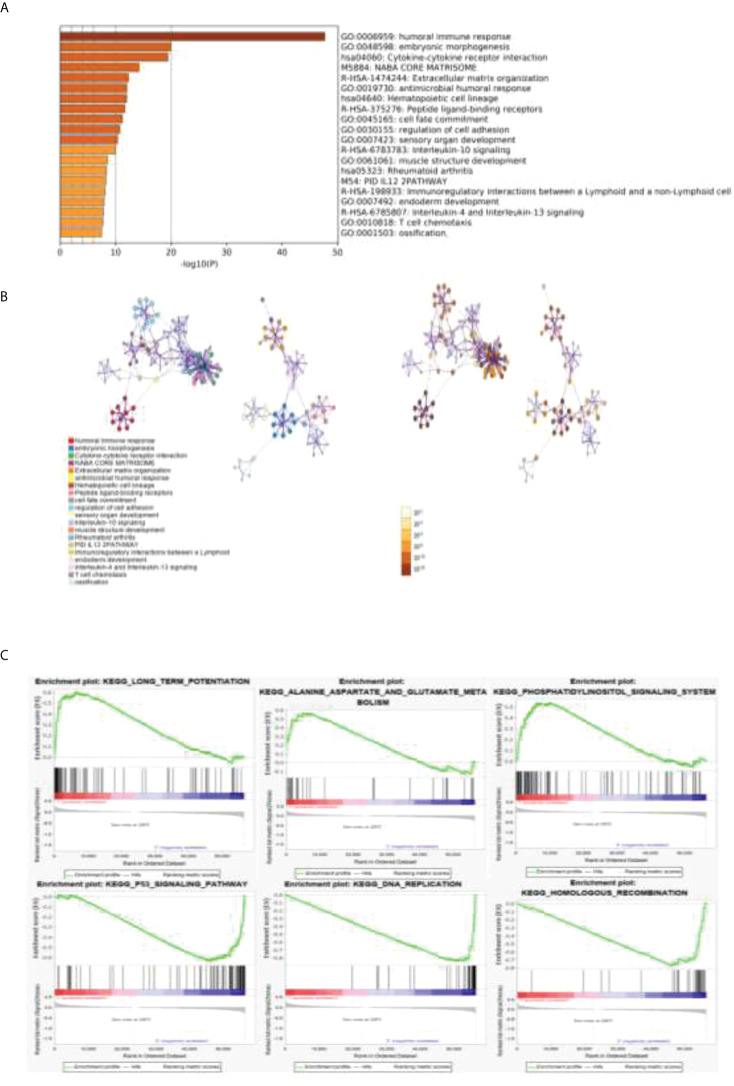
GO and KEGG analyses of related gene pathways. **(A)** DEGs about related gene pathways. **(B)** Further pathway analysis was performed by using Metascape. Each point in a pathway represents a gene. The aggregation of different color points in the left panel represents its enriched pathway, and the different color depth in the right panel represents the p-value of a different enriched pathway. **(C)** The top three pathways enriched in both clusters 1 and 2 were analyzed by GSEA enrichment analysis.

The DEGs obtained by the Metascape analysis, the darker the color value was, and the top 20 gene pathways were selected for further analysis by p-value ranking (**Figure 4A**). As shown in **Figures 4B, C**, further pathway analysis was performed using Metascape, in which each point in a pathway represents a gene, the aggregation of different color points in the left panel represents its enriched pathway, and the different color depth in the right panel represents the p-value of the enriched different pathway, and the deeper the color, the smaller the p-value.

A GSEA enrichment analysis was further performed to analyze the top three pathways enriched in both clusters 1 and 2. We found that the human immune response pathway, embryonic development pathway, and cytokine interaction pathway were the most significant. We confirmed that our results are strongly related to tumor immunity and tumor proliferation.

#### 2.2.5 Differential gene pathway analysis

**Figure 5:**
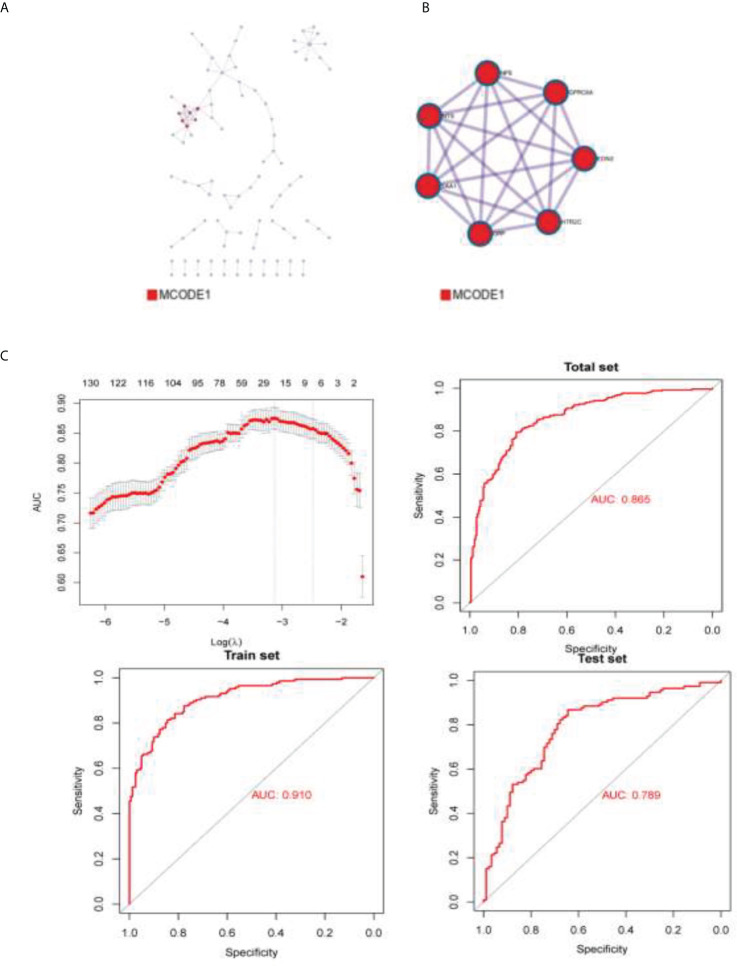
The densely connected network components in this protein interaction network. **(A)** BioGrid6, InWeb_IM7, and OmniPath8 were used to build protein–protein interaction figure. **(B)** Molecular Complex Detection algorithm was used to obtain the densely connected network components of this protein interaction network. **(C)** AUC of the prediction model.

This study conducted a lasso loop on the divergences between the two groups, and models were built with 23 differential genes by calculation (**Figures 5A, B**). After the differential genes were obtained, Metascape was used with the following databases: biogrid6, webIm7, and omnipath8 and performed the protein–protein interaction enrichment analysis. We deduced the tightly connected network components in this protein interaction network using a molecular complex detection algorithm. The cut off value is −1.96707189470124e−05. Risk score = ACP5 ∗ 0.321198235444346 + ATP6V0A4 ∗ 0.0150023269976567 + C5orf66-AS1 ∗ 0.465044242146034 + CCL3 ∗ −0.00742007191304815 + CH25H ∗ −0.0177778958861601 + COL4A2 ∗ 0.00223331121831887 + DNASE1L3 ∗ 0.0288005632566969 + EGR3 ∗ −0.0628491427983151 + ENSG00000233834 ∗ 0.409543664062472 + ENSG00000234200 ∗ 5.44120162737391 + GP9 ∗ −0.411994095719985 + HOXD11 ∗ 0.0562152633029046 ∗ IGFBP2 ∗ 0.00202071385625849 + IL1B ∗ −0.00782529664027136 + ISL2 ∗ 0.125430105679151 + LINC01602 ∗ 0.00220748348374127 + LINC02086 ∗ 0.116062428976365 + MIR5093 ∗ 0.0800999757957344 + MUC3A ∗ 2.16153840446827 + NKX2-5 ∗ 0.0229814278609885 + PRAMEF19 ∗ 4.43878690201787 + SEMG2 ∗ −3.61598536481308 + SLAMF7 ∗ 0.63875904295689. The best prediction was achieved for the classification of clusters 1 and 2. AUC (area under curve) indicates good prediction value (**Figure 5C**).

#### 2.2.6 Western blot

**Figure 6:**
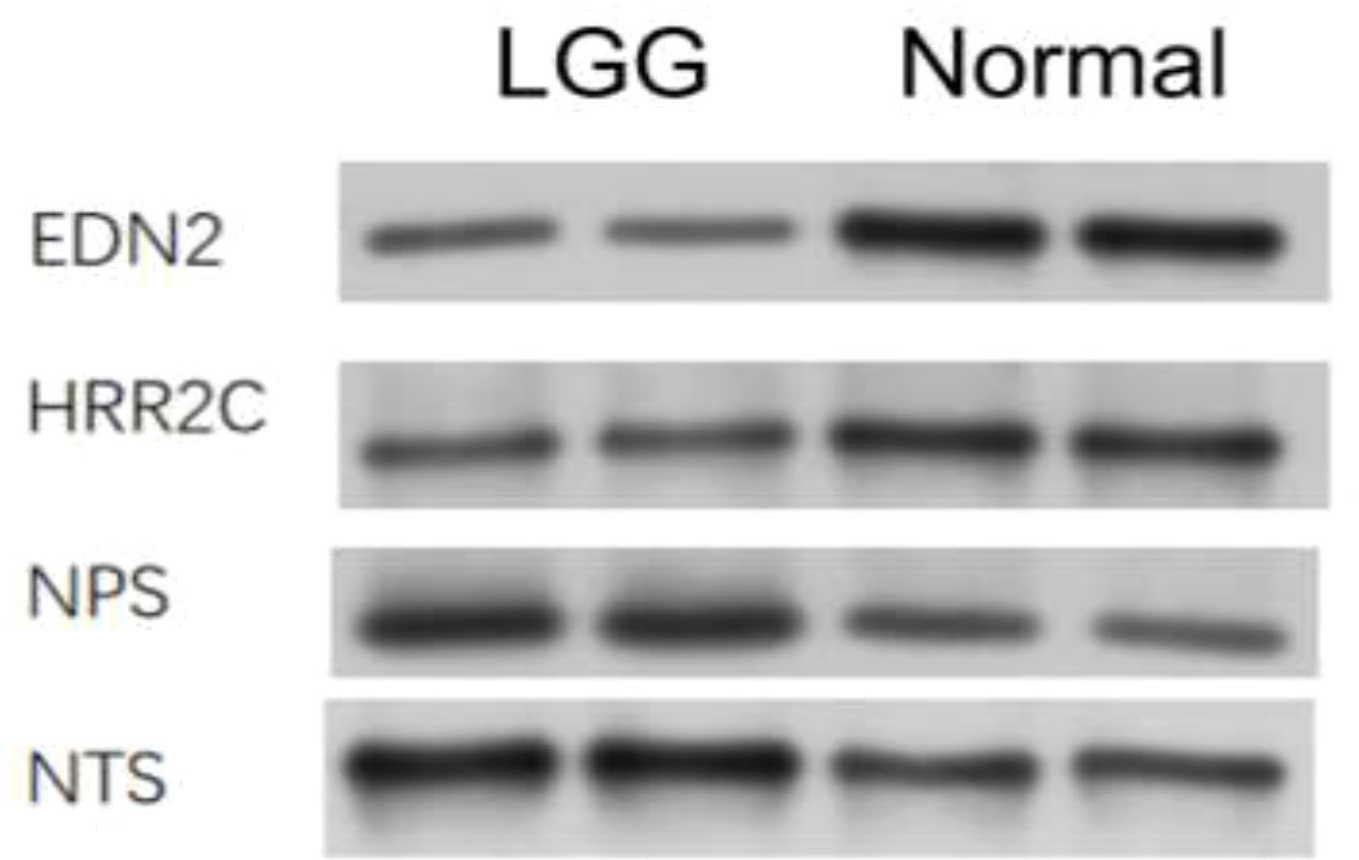
Western blot analysis on the expression of different genes in LGG cells and normal cells.

Some differential expression genes were found in the earlier analysis, so in order to confirm the differential expressions, Western blot was conducted. We took the GL261 cell line from mice and performed protein extraction after culturing with the medium for 48 h. Antibodies were bought from ABclonal Technology. The targets of Western blot are proteins expressed by genes that showed differential expression in the earlier analysis, including EDN2 (28 kd), HRR2C (75 kd), NPS (14 kd), and NTS (17 kd). The four proteins were detected in normal and tumor tissues. EDN2 and HRR2C were found to be highly expressed in normal cells. NPS and NTS were found to be highly expressed in LGG cells (**Figure 6**).

## 3 Discussion

Glioma is a disease that is difficult to treat, and patients with glioma have a poor prognosis (16, 17). This disease is also regarded as one of the most frequent primary malignancies (18, 19). The average survival rate of patients with traditional surgery combined with radiotherapy and chemotherapy is 35.7% at one year, with a five-year average survival rate of 4.7% and a median survival time of only 14.6 months. Low grade glioma contributes to one-fifth of all the glioma cases, which should be given attention. Genetic genes of tumor cells, particularly transcription factors, contribute decisively to glioma initiation and progression (20). Meanwhile, it has also been shown that much of the gene expression in tumor cells is influenced by the microenvironment of the tumor cells (21). The tumor microenvironment is the cellular environment in which the tumor resides and extracellular matrix molecules, immune cells, endothelial cells, and mesenchymal cells make up the microenvironment. The two main components of the LGG tumor microenvironment are immune cells (22) and stromal cells (23), which are widely considered to be of higher clinical value for the prognostic evaluation of tumor diagnosis than others.

To understand the relationship between immune cell infiltration and patient survival, treatment efficacy, and prognosis, data of 529 lower grade glioma cases were obtained from the TCGA for enrichment analyses. We separated tumor cells into two groups by calculating the degree of immune cell infiltration of the tumor samples to investigate the correlation of the tumor cell immune microenvironment with prognosis, clinical traits, and immunotherapy. At the same time, we developed a protein–protein interaction network by analyzing different gene expressions in two sets of tumor cell samples and exploring pathways for subsequent studies. This article hopes to reveal the relationship between the degree of immune infiltration and patient survival, therapeutic efficacy, as well as prognosis through the analysis of numerous tumor samples, and to seek the relationship between gene expression and the immune microenvironment of tumor cells by analyzing different protein–protein interaction networks within LGG cells, which are also analyzed in this paper, to find valuable pathways in preparation for further studies. From the electrophoretic stripe, it is obvious that all targeted protein content is higher in LGG cells than in normal tissue. Our Western blot results indicate the existence of certain genes and provide strong practical evidence for the database analysis.

This experiment has high clinical value. The experimental tumor samples were divided into two groups, cluster 1 and cluster 2, depending on the immune microenvironment infiltration. Overall survival was higher for cluster 1 than for cluster 2. Analysis of the immune infiltration of samples from both groups revealed significant differences in the expression of macrophages m2, monocytes, resting memory CD4 T cells, and differences in immune cells at the sites examined. Prediction of immunotherapy effects was performed on samples by TIDE (an online prediction tool), and TIDE values were obtained. A TIDE value greater than 0 and higher the sensitivity to immunotherapy such as PD1, less than 0 and lower. As can be seen, this experiment concluded the sensitivity of tumor cell genes to immunotherapy by quantitative analysis and predicted the effect of immunotherapy, which is extremely instructive for practical clinical operations.

In this experiment, the gene expression of tumor cells was analyzed. The edger was used to find the differential gene expression between the two groups of samples with different immune microenvironments, and we developed a protein–protein interaction network using pathway analysis and obtained the densely connected network components in the protein interaction network. The models constructed with differential genes had excellent predictive effects for the classification of cluster 1 and cluster 2, demonstrating the relationship between the differential expression of tumor cell genes and the tumor microenvironment. Clinically, it is possible to predict therapeutic effects by analyzing the genetic makeup of tumor cells of a patient to understand the state of the tumor cell microenvironment.

The present experiments also present inadequate and worthwhile aspects of in-depth study. Tests such as alignment with data from genes within other databases or the introduction of a scoring system to score the immune microenvironment and classify samples according to the score can make experiments more precise. Differential genes can be studied in more depth, pointing to mechanisms of action between the immune microenvironment and gene expression in more detail.

In conclusion, our current study has strong practical value for predicting the efficiency of immunotherapy as well as the prognosis of LGG patients, and it lays the foundation for further uncovering the potential associations among differential gene expression, immune microenvironment, and prognosis of LGG patients.

## Data availability statement

The original contributions presented in the study are included in the article/supplementary material. Further inquiries can be directed to the corresponding authors.

## Author contributions

WeiJ and ZH conceptualized the study. WeizJ and CL prepared the experiment. JD, CL and LY prepared the figures and tables. WeizJ and XL wrote the introduction and methods. ZH and FX wrote other parts of the article. All authors listed have made a substantial, direct, and intellectual contribution to the work and approved it for publication.

## Funding

This work was supported by the 2020 Fund Project of the Shanghai Health Committee under grant No. 202040106.

## Conflict of interest

The authors declare that the research was conducted in the absence of any commercial or financial relationships that could be construed as a potential conflict of interest.

## Publisher’s note

All claims expressed in this article are solely those of the authors and do not necessarily represent those of their affiliated organizations, or those of the publisher, the editors and the reviewers. Any product that may be evaluated in this article, or claim that may be made by its manufacturer, is not guaranteed or endorsed by the publisher.
